# Single-molecule fluorescence imaging techniques reveal molecular mechanisms underlying deoxyribonucleic acid damage repair

**DOI:** 10.3389/fbioe.2022.973314

**Published:** 2022-09-15

**Authors:** Yujin Kang, Soyeong An, Duyoung Min, Ja Yil Lee

**Affiliations:** ^1^ Department of Biological Sciences, Ulsan National Institute of Science and Technology, Ulsan, South Korea; ^2^ Department of Chemistry, Ulsan National Institute of Science and Technology, Ulsan, South Korea; ^3^ Center for Genomic Integrity, Institute of Basic Sciences, Ulsan, South Korea

**Keywords:** single-molecule technique, fluorescence imaging, real-time visualization, DNA damage, DNA repair mechanism

## Abstract

Advances in single-molecule techniques have uncovered numerous biological secrets that cannot be disclosed by traditional methods. Among a variety of single-molecule methods, single-molecule fluorescence imaging techniques enable real-time visualization of biomolecular interactions and have allowed the accumulation of convincing evidence. These techniques have been broadly utilized for studying DNA metabolic events such as replication, transcription, and DNA repair, which are fundamental biological reactions. In particular, DNA repair has received much attention because it maintains genomic integrity and is associated with diverse human diseases. In this review, we introduce representative single-molecule fluorescence imaging techniques and survey how each technique has been employed for investigating the detailed mechanisms underlying DNA repair pathways. In addition, we briefly show how live-cell imaging at the single-molecule level contributes to understanding DNA repair processes inside cells.

## 1 Introduction

**FIGURE 1:**
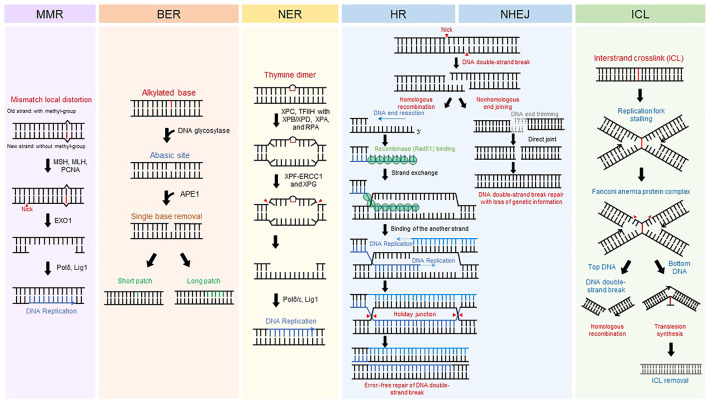
DNA repair mechanisms. Representative eukaryotic DNA repair pathways and their associated proteins. Schematic procedure of each eukaryotic DNA repair pathway is displayed.

All living organisms on earth maintain themselves by transferring their genetic information to offspring through DNA replication. Preservation of DNA, the genetic material, is important for sustaining life. Even in normal circumstances, however, DNA is injured by intracellular metabolites such as radical oxygen species and environmental factors including ultraviolet (UV) from the Sun and pollutants (Friedberg Errol C, 2005). Damaged DNA is immediately restored by DNA repair systems that are evolutionarily conserved. Different types of DNA repair machinery are activated according to types of DNA damage (Friedberg Errol C, 2005) (Figure 1). Base mismatches due to errors in replication are corrected by the mismatch repair (MMR) mechanism (Jiricny, 2006; Pecina-Slaus et al., 2020). Base modifications from alkylated agents such as oxidized guanine are removed by base excision repair (BER) (Wallace, 2014; Beard et al., 2019). UV-induced pyrimidine dimers and bulky chemical adducts to bases are repaired by nucleotide excision repair (NER) (Scharer, 2013; Marteijn et al., 2014). DNA double-strand break (DSB), the most fatal type of damage, is repaired by homologous recombination (HR) in an error-free manner or non-homologous end joining (NHEJ) that fixes DSBs in an error-prone manner (Chang et al., 2017; Kaniecki et al., 2018). Interstrand crosslinks (ICLs) causing replication stalling are resolved by the Fanconi anemia repair pathway (Ceccaldi et al., 2016). In addition to these repair processes, other repair mechanisms safeguard against genomic instability. Malfunction of DNA repair systems causes malignant diseases (Jackson and Bartek, 2009). Defective MMR causes colon cancer and hereditary non-polyposis colorectal cancer. BER is related to neural disorders and cancer. Failure of NER can induce UV-sensitive skin problems, xeroderma pigmentosum, and, more seriously, skin cancers. HR is closely associated with breast and ovarian cancers. Hence, delving into the molecular mechanisms underlying DNA repair is crucial for better understanding human diseases and improving therapeutic interventions.

Traditional biochemical approaches are limited in their ability to closely examine molecular interactions due to intrinsic biological heterogeneity. This limitation is removed by observing molecules individually (Joo et al., 2008). Single-molecule techniques not only unravel molecular substates hidden by ensemble-average effects but also enable precise estimation of kinetics in biomolecular reactions. Since the single-molecule concept emerged approximately 30 years ago, diverse single-molecule methods have been developed to unravel numerous molecular details that would otherwise have been veiled (Joo et al., 2008; Neuman and Nagy, 2008; Li et al., 2020). In particular, single-molecule fluorescence imaging methods have been widely used to probe DNA metabolic reactions, including DNA damage repair, and have provided an abundance of indirect and direct evidence (Joo et al., 2008). Despite the great progress and their own advantages, however, it is noted that there are several downsides that demand further evolution. Single-molecule flow cell preparation still remains labor intensive tasks. Biomolecules are easily/nonspecifically absorbed onto the cell surface, demanding exhaustive surface passivation (Paul et al., 2021). Besides, viable concentrations of dye-labeled molecules are limited up to tens of μM for most assays due to the background noise (Goldschen-Ohm et al., 2017). Photobleaching of dyes further limits long-time watching of single-molecules. Even with all the constraints, however, it is no doubt that the single-molecule approaches enable unprecedented, detailed observations masked by ensemble averaging.

In this paper, we introduce representative single-molecule fluorescence imaging techniques and survey their utilization for unveiling the molecular mechanisms underlying DNA damage repair pathways.

## 2 Protein-induced fluorescence enhancement

**FIGURE 2:**
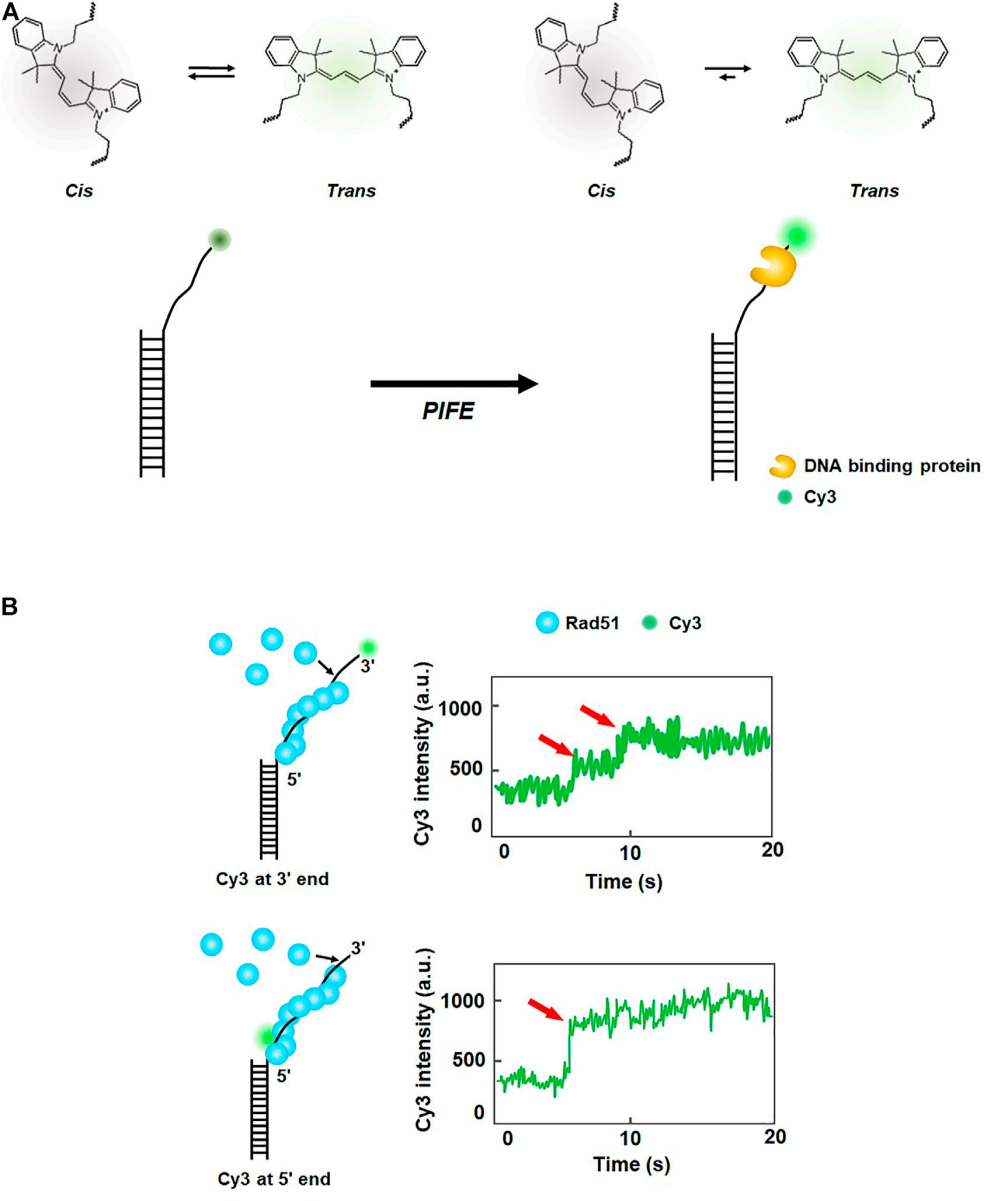
Protein-induced fluorescence enhancement (PIFE). **(A)** Principle of PIFE. In the absence of a protein, the isomers of Cy3 interconvert between *cis* and *trans* states. When a DNA-binding protein is placed in the vicinity of Cy3, interaction with the protein induces transition to the *trans* state, which has a higher quantum yield than the *cis* state and enhances fluorescence intensity. **(B)** Fluorescence signal change according to the labeling position of Cy3 in ssDNA. When Cy3 is labeled at the 3′ end of ssDNA, fluorescence intensity increases in multiple steps, while one-step increase in fluorescence is observed when Cy3 is internally tagged at the 5′ end of ssDNA, demonstrating that growth of the Rad51 filament proceeds from the 3′ to 5′ end of ssDNA. Fluorescence intensity signals are depicted based on the reference (Qiu et al., 2013).

The excited state of some fluorescent dyes such as Cy3 exists in either of two isomers, *cis* or *trans*. The *trans* state has a higher quantum yield than the *cis* state (Figure 2A). At equilibrium, the two isomers are interconverting. However, when a protein is placed in the vicinity of Cy3, the interaction between the protein and Cy3 shifts equilibrium toward the *trans*-state, and hence the fluorescence intensity of Cy3 increases. This phenomenon is known as protein-induced fluorescence enhancement (PIFE) (Hwang et al., 2011) (Figure 2A). PIFE is applicable for interactions between an unlabeled protein and fluorescently labeled nucleic acids (Hwang and Myong, 2014). For example, PIFE can either be used for determining whether a protein binds a target site, or to measure the distance between a protein binding position and a target sequence. Because PIFE does not need to label the target protein with a fluorophore, it avoids the labeling effect that might disturb protein activity.

In eukaryotic HR, Rad51 binds single-stranded DNA (ssDNA) resected from a broken DNA end and forms a presynaptic filament that searches for a homologous sequence and exchanges strands. Using the single-molecule PIFE assay, Qiu *et al.* examined the directionality of presynaptic filament formation of yeast Rad51. In a DNA construct with a poly dT (dT_20_) single-stranded overhang and biotinylated duplex stem, Cy3 was labeled at either 3′ or 5′ end of the overhang (Qiu et al., 2013) (Figure 2B). The DNA was anchored on a PEGylated slide surface via biotin-neutravidin linkage. For 3′ end Cy3 labeling, the addition of Rad51 led to a multi-step fluorescence increase, whereas a single-step fluorescence increase was observed for 5′ end Cy3 labeling (Figure 2B). The rate of fluorescence increase for 5′ end labeling was 3.3 times higher than that for 3′ end labeling. Taken together, these results demonstrate that the filament formation proceeds from the 5′ end to the 3’ end of ssDNA.

Microwell PIFE (mwPIFE) integrates PIFE into a microwell plate fluorescence reader (Valuchova et al., 2016). MwPIFE is a rapid, cost-effective, and high-throughput platform for a wide range of applications for protein-DNA interactions. In mwPIFE, Cy3-labeled biotinylated DNA is anchored to the surface of neutravidin-coated microwells. MwPIFE was applied for determining the relative binding specificity of XPF-ERCC1, which is involved in multiple DNA repair pathways as an endonuclease. PIFE was measured for a 10-nt ssDNA overhang in the presence of diverse DNA substrates such as hairpin, fork, duplex, and ssDNA as competitors. The relative binding affinity of XPF-ERCC1 to each DNA substrate was estimated. These experiments show that XPF-ERCC1 acts on branched DNA structures with a junction of double-stranded DNA (dsDNA) and ssDNA including long mismatches (e.g., bubbles) and forks. Moreover, mwPIFE was used to examine interactions between DNA and the Ku70/80 complex, which has a high binding affinity to the end of duplex DNA (Valuchova et al., 2016). In the mwPIFE assay, the binding affinity of the Ku complex was dramatically reduced when both ends of duplex DNA were blocked by neutravidin. These results demonstrate that the free end of duplex DNA is essential for the binding of Ku70/80, and at least 13–15 bp are required for binding of the Ku complex at the DNA end, consistent with a previous study (Yoo et al., 1999). The authors also observed translocation of the Ku complex along duplex DNA with internally labeled Cy3 using mwPIFE.

## 3 Single-molecule fluorescence resonance energy transfer

**FIGURE 3:**
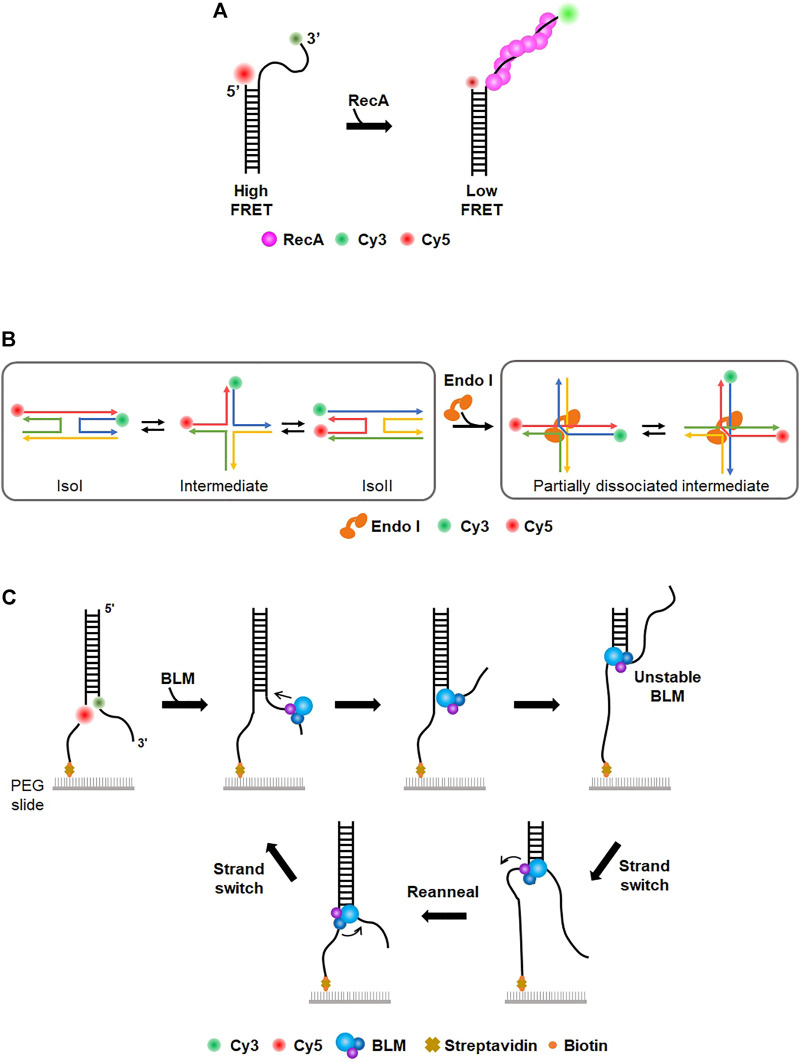
Single-molecule FRET (smFRET). **(A)** Study of RecA filament formation using smFRET. Donor (Cy3) and acceptor (Cy5) dyes are labeled at the 3′ end of the ssDNA overhang and junction, respectively. In the absence of RecA, the flexibility of ssDNA overhang allows high FRET. The addition of RecA forms a nucleoprotein filament on ssDNA, which extends ssDNA and induces low FRET. **(B)** Conformational dynamics of Holliday junction (HJ) and the effect of HJ-resolving enzymes (Endo I). (Left) The four-way junction has two conformers (isoI and isoII) that can be distinguished by low and high FRET efficiency, respectively. The conformational dynamics of HJ are characterized by smFRET (Right) HJ-resolving enzyme (e.g., EndoI). induces a partially dissociated intermediate, which is evolutionarily conserved. **(C)** Donor (Cy3) and acceptor (Cy5) dyes are internally labeled at the junction of the fork structure to allow high FRET. BLM helicase translocates from the 3′ to 5′ end and unwinds dsDNA, lowering FRET efficiency. After BLM separates a certain length of duplex DNA, BLM transfers to the other strand and reanneals the duplex. This strand switching happens repetitively.

Fluorescence resonance energy transfer, or Förster resonance energy transfer (FRET), is one type of energy transfer through dipole-dipole interaction between two fluorescent dyes, donor and acceptor. The donor has a shorter emission wavelength (i.e., higher emission energy) than the acceptor. Through FRET, the acceptor dye emits fluorescence only when the donor dye is excited. The energy transfer efficiency is inversely proportional to the sixth power of the distance between the two dyes. When the two dyes are far apart, only the donor fluoresces, whereas the acceptor emits fluorescence when dyes are close to each other. In general, FRET occurs when the two dyes are <10 nm apart and hence is considered as a nanometric ruler. Single-molecule FRET (smFRET) measures FRET signals for individual molecules. Currently, the smFRET technique is widely used for studying conformational dynamics for a variety of biomolecules (Lerner et al., 2018). Taekjip Ha, a pioneer of smFRET, intensively studied prokaryotic HR processes (Joo et al., 2006; Roy et al., 2009; Park et al., 2010). When DNA DSB occurs in *E. coli*, end resection by RecBCD, which is a helicase complex with nuclease activity, initiates the HR pathway (Bianco et al., 2001; Dillingham and Kowalczykowski, 2008; Smith, 2012). ssDNA binding proteins (SSBs) bind to the resected tail and are subsequently replaced by recombinase RecA to form a presynaptic filament (Dixon and Kowalczykowski, 1991; Spies and Kowalczykowski, 2006). Using smFRET, formation of a RecA filament on a 30-nt ssDNA tail was observed (Joo et al., 2006) (Figure 3A). The donor (Cy3) and acceptor (Cy5) fluorophores were labeled at the end of the ssDNA tail and ss-dsDNA junction, respectively. While the flexibility of ssDNA gave high FRET in the absence of RecA, the addition of RecA changed FRET efficiency from high to low state, indicating that ssDNA was straightened by RecA filament formation. They estimated the binding affinity of RecA at the 5′ end (K_d_: ∼100 nM) and 3′ end (K_d_: ∼8 nM) of ssDNA, suggesting that the directionality in the filament elongation is mainly due to the difference in the binding rates. They also observed that SSB was replaced by RecA on ssDNA (Joo et al., 2006). In the absence of RecA, ssDNA was coated with SSB, which structurally shortened the end-to-end distance of ssDNA and gave high FRET. However, when RecA was added after unbound SSB was washed out, the FRET signal became low, showing that RecA replaces SSB and the ssDNA is extended by RecA filament. Next, how SSB can be displaced by RecA was addressed (Roy et al., 2009). Binding of SSB to 65–70-nt ssDNA, which had donor (Cy3) and acceptor (Cy5) fluorophores at either end, produced a high FRET state because both ends of ssDNA become structurally close on SSB. When several nucleotides were added to ssDNA, FRET efficiency fluctuated, implying that SSB diffuses on ssDNA (Roy et al., 2009). FRET data also showed that the size of the diffusion step of SSB is ∼3 nts, which is identical to the binding site size of RecA monomer (Chen et al., 2008; Roy et al., 2009). Moreover, three-color FRET was used for elongation of the RecA filament followed by SSB diffusion and dissociation. Single donor (Cy3) was internally labeled in ssDNA, and two acceptor dyes (Cy5 and Cy5.5) were labeled at the 3′ and 5′ ends of ssDNA, respectively. When SSB bound to ssDNA, RecA addition reduced FRET between Cy3 and Cy5.5 because RecA assembles from the 5’ end of ssDNA. The Cy5 fluorescence burst out due to FRET between Cy3 and Cy5, indicating that SSB diffuses along with RecA filament growth. In addition, the authors found that SSB diffusion could melt a secondary structure such as a hairpin that might be formed in ssDNA and promoted RecA filament growth.

In addition to prokaryotic HR, eukaryotic HR mechanisms were also examined by smFRET. Human Rad52 (hRad52) is a recombination mediator that promotes annealing of complementary strands in homology-directed DNA repair (Symington, 2002; Sugawara et al., 2003; Rothenberg et al., 2008; Grimme et al., 2010). To examine hRad52 activity in DNA annealing, two partially complementary ssDNA molecules were labeled with donor and acceptor, respectively (Rothenberg et al., 2008). The addition of hRad52 produced a high FRET signal, indicative of the annealing of two complementary ssDNA molecules by hRad52. During the annealing process, the initial FRET signal was unstable, but the FRET trace was not discontinuous, implying that initial pairing by hRad52 occurred between regions that were not fully complementary and that hRad52 searched for complementary regions to anneal two ssDNAs. smFRET revealed that hRad52 mediates the annealing of replication protein A (RPA)-coated ssDNA via an interaction between hRad52 and RPA (Grimme et al., 2010). In eukaryotic HR, the Mre11/Rad50/Nbs1 (MRN) complex is an essential factor for signaling responses of DSB as well as DNA end resection-initiating HR (Williams et al., 2007; Paull, 2010). MRN binding and unwinding at the end of duplex DNA were monitored in real-time (Cannon et al., 2013), showing that MRN promotes local separation (∼15–20 bp) of DNA strands in an ATP-dependent manner. It was also shown that smFRET is a suitable tool for characterizing the dynamics of the Holliday junction (HJ), which is a structural intermediate in HR (Lilley, 2000). The conformational dynamics and branch migration of HJ were well established using smFRET (McKinney et al., 2003; Lee et al., 2005) (Figure 3B). HJ had two conformers, isoI and isoII, which were distinguished by FRET efficiency. The transition between the two conformers was influenced by sequence and divalent cation. In addition, how HJ-resolving enzymes affect HJ dynamics and branch migration was examined. Several HJ-resolving enzymes from different organisms were tested (Zhou et al., 2019). When the enzymes bound to HJ, a partially dissociated intermediate was commonly formed, allowing unencumbered dynamics in HJ conformational changes and branch migration (Figure 3B).

On the other hand, helicases play pivotal roles in various DNA metabolic reactions, including DNA replication and repair. smFRET was used to characterize the mechanistic properties of helicases in duplex unwinding and motor activity. Paul *et al.* investigated the unwinding of human telomeric G-quadruplex (G4) by Rep helicase, which is a super-family I (SF1) helicase in DNA replication (Paul et al., 2020). In the smFRET assay, stable G4 displayed high FRET efficiency due to its compact structure. When Rep helicase was added to G4, FRET efficiency decreased over time, demonstrating that Rep helicase unwinds the G4 structure. This suggests that Rep helicase is a new factor to resolve deleterious G4 structures in DNA replication (Paul et al., 2020). *B. stearothermophilus* PcrA, as a non-replicative 3′-5′ SF1 helicase, is known to remove RecA at stalled replication forks. The molecular activity of PcrA was recapitulated by smFRET (Anand et al., 2007; Park et al., 2010). The pair of donor and acceptor dyes were labeled at several different locations of DNA. The FRET efficiency of each pair showed that PcrA induces looping of a 5′ single-stranded tail and preferentially translocated on the lagging strand instead of unwinding the template duplex. When PrcA was added to the RecA filament on ssDNA, FRET efficiency increased, indicating that PcrA removes RecA from ssDNA. These findings reveal a novel mechanism for eliminating toxic recombination intermediates at stalled replication forks. Bloom syndrome helicase (BLM) is an ATP-dependent 3′-5′ helicase involved in HR and replication (Karow et al., 1997). The unwinding activity of BLM was scrutinized using smFRET with donor and acceptor dyes at each strand showing high FRET (Yodh et al., 2009). Surprisingly, BLM repetitively unwound individual duplex DNA even in the presence of RPA. This repetitive unwinding was possible because BLM switched the strand and reannealed the DNA after it unwound a short length (<34 bp) of duplex DNA from the 3′ to 5′ end (Figure 3C). XPD is a helicase involved in prokaryotic NER that unwinds lesion-containing strands (Liu et al., 2008). During strand separation, XPD inevitably collides with RPA, which preferentially binds to unwound ssDNA. How XPD deals with RPA was studied using smFRET (Honda et al., 2009). RPA and ssDNA were labeled with acceptor and donor dyes, respectively, displaying high FRET without XPD. The addition of *F. acidarmanus* XPD changed the conformational dynamics of RPA-coated ssDNA, producing two modes of XPD translocation along RPA-coated ssDNA, RPA dissociation from ssDNA and translocation along ssDNA over the RPA without displacement.

The smFRET technique has been used to understand the MMR pathway. Msh2-Msh3, which serves as a sensor of long mismatches, has lower repair efficiency for (CAG)_7–13_ hairpins than for (CA)_4_ loops. The mechanism behind this loop discrimination by Msh2-Msh3 was elucidated by smFRET (Lang et al., 2011). In three-way junction DNA, both donor and acceptor dyes were labeled at one strand, and the other strand contained a (CA)_4_ loop or (CAG)_7–13_ hairpin in the center. The binding of Msh2-Msh3 to (CA)_4_ loop DNA slightly increased FRET efficiency due to the bending of the (CA)_4_ loop by Msh2-Msh3. By contrast, for (CAG)_13_ hairpin DNA, the addition of Msh2-Msh3 resulted in a marginally lower FRET state in addition to a higher FRET state. The lower FRET state disappeared when the stem sequences of the (CAG)_13_ hairpin were changed to form a duplex. These results suggest that the binding of Msh2-Msh3 to (CAG)_13_ hairpins generates another discrete conformational state that is different from bending formation, which produces a lower FRET state and prevents Msh2-Msh3 from proceeding to next steps (Lang et al., 2011).

Two-color smFRET was evolved into four-color smFRET to tackle complex biological systems (Lee J. et al., 2010). The conformational dynamics of HJ were dissected by the four-color smFRET technique, in which Cy2, Cy3, Cy5, and Cy7 are labeled at each end of the four arms. Four-color smFRET allows “dual FRET pair” measurement, in which two independent FRET pairs simultaneously measure the correlation. The dual FRET pair system was used to examine RecA-mediated strand exchange. Cy5 and Cy7 were labeled at the junction and 3’ single-stranded tail of a primer-template junction as acceptors. The single-stranded tail was coated with RecA. Duplex DNA that was homologous to the single-stranded tail was labeled with two donors, Alexa488 and Cy3. When the homologous duplex DNA reacted with the RecA filament, three types of FRET traces were observed: 1) no delay between docking and completion of strand exchange, 2) a delay at only one of the two ends, and 3) a delay at both ends. These results demonstrate that strand exchange initiates from either end or the middle of DNA. The four-color smFRET system enables elaborate observations that cannot be made using conventional two-color FRET.

## 4 DNA combing

**FIGURE 4:**
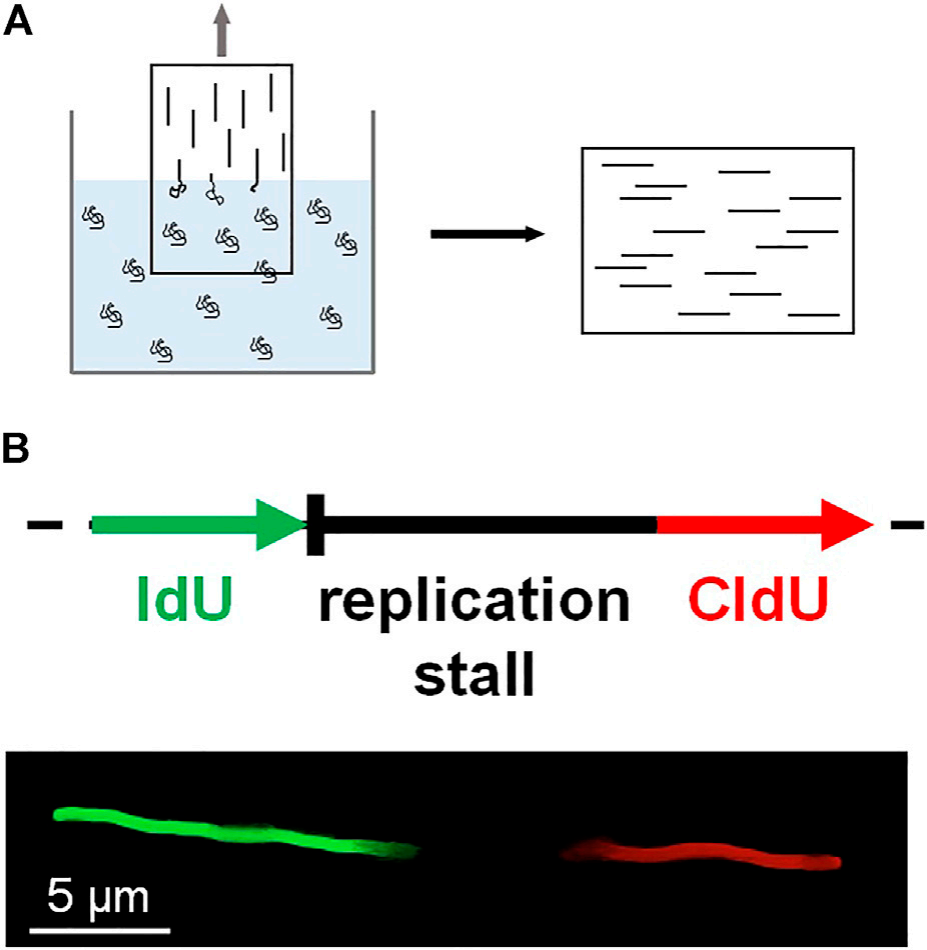
DNA combing. **(A)** A coverslip coated with positively charged polymers is slowly pulled out of a solution containing DNA molecules. DNA becomes stretched at the air-water interface and sticks to the coverslip surface. The stretched DNA molecules are stained and fluorescently imaged. **(B)** Schematic of DNA fiber assay based on DNA combing. During DNA replication, IdU (green) is incorporated into DNA before replication fork stalling, whereas CldU (red) is treated after fork stalling. The length of the nascent strand before fork stalling is estimated by the length of the IdU track.

DNA combing assay is an early method of single-molecule imaging based on the surface stretching of DNA molecules (Michalet et al., 1997; Gueroui et al., 2002; Bianco et al., 2012). The surface of a glass coverslip is modified with positively charged polymers. The coverslip is dipped into a solution containing DNA molecules, which are randomly tethered to the surface. Then the coverslip is slowly pulled out of the solution, and surface-tethered DNA molecules become stretched at the water-air meniscus (Figure 4A). Individual stretched DNA molecules are stained and measured using fluorescence microscopy. This technique is also known as ‘DNA fiber assay’ to study replication stress, which causes DNA damage and genomic instability (Techer et al., 2013; Nieminuszczy et al., 2016; Betous et al., 2018). Schlacher *et al.* investigated the role of BRCA2 at stalled replication forks (Schlacher et al., 2011). BRCA2, a key player in DSB repair, promotes and stabilizes Rad51 nucleoprotein filament formation on RPA-coated ssDNA (Chatterjee et al., 2016). In the DNA combing assay, IdU or CldU is incorporated into newly synthesized DNA, and IdU tracks represent nascent DNA strands before replication fork is stalled (Figure 4B). IdU tracks were shorter in BRCA2-deficient cells than in wild-type cells, implying that BRCA2 mediates the protection of nascent strands at stalled replication forks. The DNA combing assay also showed that the interaction between Rad51 and the BRC motif in the C-terminus of BRCA2 is crucial for fork protection. For further protection of replication forks, BRCA2 blocked the nucleolytic activities of Mre11 (Schlacher et al., 2011).

DNA combing assay was also used to explore coordination between the NER pathway and checkpoint pathway by Exo1, which has both 5′-3′ exonuclease and 5′ flap endonuclease activity (Giannattasio et al., 2010). For UV-exposed DNA, NER generates a ∼30-nt ssDNA gap, which is originally refilled by replication and ligation. The DNA combing assay showed that ssDNA gaps longer than 30-nt were generated by UV irradiation in noncycling cells but not in Exo1-deficient cells. Moreover, in the case that the gap-refilling reaction was suppressed, Exo1 extended the ssDNA gap and promoted Mec1 kinase activation and long-patch repair synthesis. Collectively, these results provide evidence that alternative repair pathways can be activated when a DNA repair pathway is problematic (Giannattasio et al., 2010).

## 5 DNA curtain

### 5.1 Single-tethered DNA curtain

**FIGURE 5:**
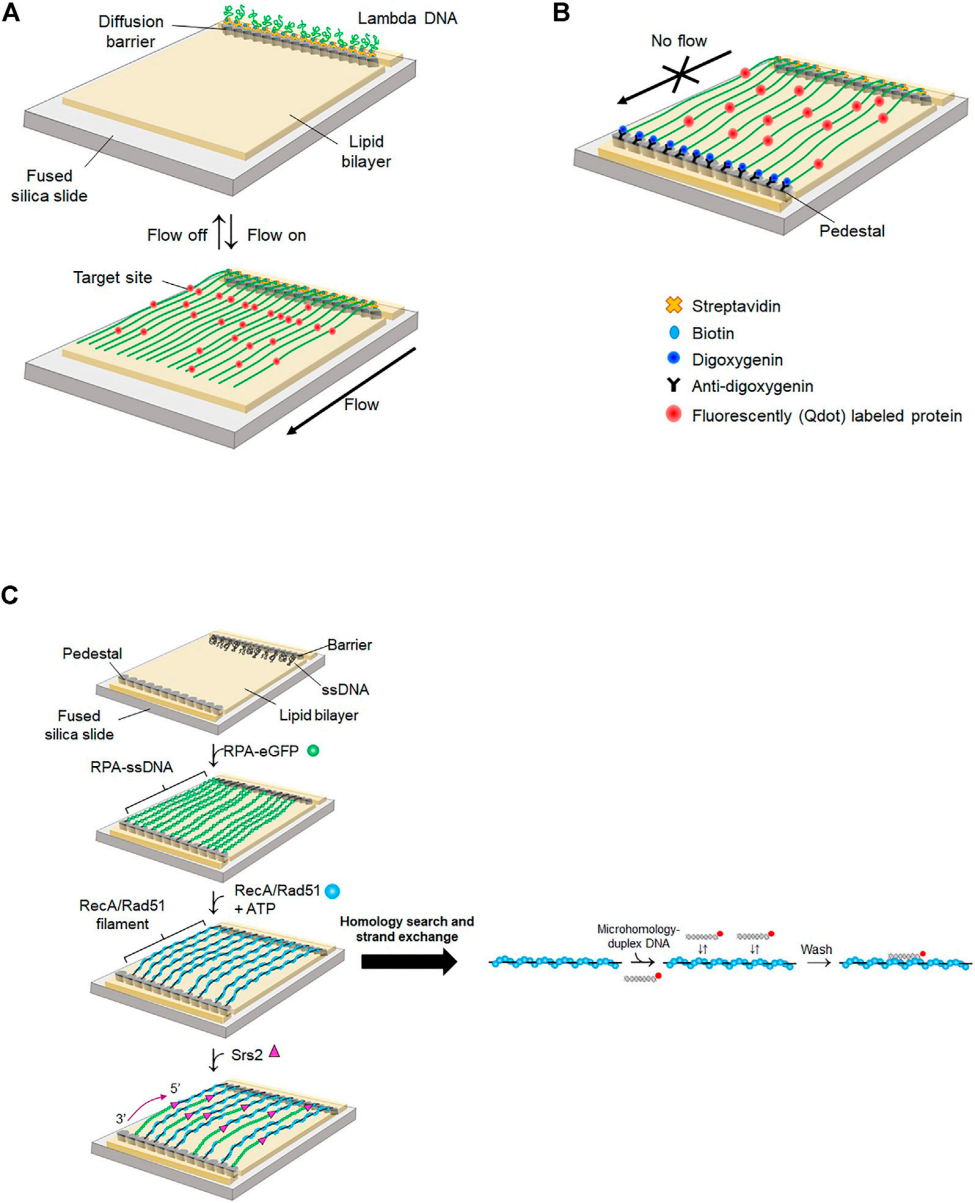
DNA curtain. **(A)** Single-tethered DNA curtain (ST-DNA curtain). One end of lambda DNA is anchored on a lipid molecule via biotin-streptavidin linkage and the other end is free. When buffer flow is switched off, DNA is flexibly coiled, whereas DNA is stretched and aligned at a diffusion barrier in the presence of buffer flow that exerts a hydrodynamic shearing force to DNA. Proteins are labeled with Qdot. Both DNA and proteins are imaged by TIRFM. **(B)** Double-tethered DNA curtain (DT-DNA curtain). The other end of lambda DNA is tagged with digoxigenin and tethered to a nanometric pedestal, on which anti-digoxigenin is adsorbed. DNA remains stretched in the absence of buffer flow. Proteins are labeled with Qdot. Both DNA and proteins are imaged by TIRFM. **(C)** Single-stranded DNA curtain. Long ssDNA generated by rolling circle amplification is anchored on a lipid bilayer through biotin-streptavidin interaction. ssDNA molecules are extended and fluorescently imaged by RPA-eGFP. In the absence of free RPA-eGFP, recombinase such as RecA or Rad51 replaces RPA from ssDNA. Homology search and strand exchange are examined by dissociation of fluorescently labeled microhomology-containing duplex DNA from the presynaptic complex. Srs2, an anti-recombinase, detaches Rad51 from ssDNA and promotes re-association of RPA-eGFP from the 3′ end of ssDNA.

Eukaryotic DNA is packaged into compact chromatin in a nucleus. Even though super-resolution imaging techniques allow the visualization of individual proteins, monitoring the dynamic behavior of proteins inside a nucleus is still challenging due to restricted spatiotemporal resolution. Instead, the motion of proteins on DNA and protein-DNA interactions are imaged *in vitro* by stretching DNA molecules on a surface (Kabata et al., 1993; Jarrous and Reiner, 2007). DNA curtain assay is a high-throughput platform integrating lipid fluidity, microfluidics, and total internal reflection fluorescence microscopy (TIRFM) (Kang et al., 2022). Biotinylated lambda phage DNA molecules are anchored on a biotinylated lipid bilayer via streptavidin. Hydrodynamic flow exerts a shearing force on DNA molecules, which move on the lipid bilayer due to lipid fluidity and are stuck at a diffusion barrier made of nanometer-sized chromium. As only one end of DNA is anchored on the bilayer, this configuration is called a ‘single-tethered DNA curtain’ (ST-DNA curtain) (Figure 5A). ST-DNA curtain is suitable for investigating DNA-end binding proteins because the other end of DNA is free. Therefore, the ST-DNA curtain has been employed for studying DSB repair pathways such as HR and NHEJ (Finkelstein et al., 2010; Stinson and Loparo, 2021). Using ST-DNA curtain, Finkelstein *et al.* examined the behavior of RecBCD during DNA end resection (Finkelstein et al., 2010). ST-DNA curtain directly visualized the DNA end resection process of RecBCD. The speed and processivity of RecBCD were accurately quantified. The speed change of RecBCD when it encountered the Chi sequence was observed. Furthermore, the authors elucidated how RecBCD deals with protein obstacles during end resection. RecBCD and DNA-binding proteins were labeled with different quantum dots (Qdots). When RecBCD collided with RNA polymerase, nucleosomes, or EcoRI-catalytic mutants (EcoRI^E111Q^), which still have high binding affinity to nonspecific sites, the protein obstacle dissociated while being pushed by RecBCD, suggesting that the protein obstacle is eliminated by RecBCD when it reaches a transition state, in which its binding affinity is low. By contrast, the Lac repressor spontaneously dissociated from DNA when it was pushed to nonspecific sequences by RecBCD, as the Lac repressor has low binding affinity for nonspecific sequences.

When DSBs occur in eukaryotes, poly (ADP-ribose) polymerase-1 (PARP-1) arrives first at the damage and synthesizes ADP-ribose polymer, which then decondenses local chromatin and recruits DNA repair enzymes (Caron et al., 2019). In ST-DNA curtain, PARP-1 diffused along DNA, showing that PARP-1 searches for DNA damage or broken ends via 1D diffusion. In addition, PARP-1 pre-bound to the end of DNA blocks Exo1 binding, indicating that PARP-1 masks broken ends and suppresses DNA end resection by Exo1.

In eukaryotes, DSBs are repaired by HR or NHEJ. Which pathway is chosen depends on the phase of the cell cycle, with DNA end resection playing a decisive role in the choice. NHEJ is initiated from DSB recognition of Ku70/80 heterodimer, whereas HR-mediated repair is predominant when broken ends are resected mostly in S-phase (Deshpande et al., 2020; Oz et al., 2021). DNA-dependent protein kinase (DNA-PK) consists of DNA-PKcs (a DNA-dependent protein kinase catalytic subunit) and Ku70/80 and binds broken ends of dsDNA during all phases including S-phase (Chanut et al., 2016). How DNA-PK regulates DNA end resection and promotes HR in S-phase was elucidated using ST-DNA curtain (Deshpande et al., 2020). The phosphorylation of Ku70/80 by DNA-PKcs reduced the lifetime of DNA-PK (the complex of Ku70/80 and DNA-PKcs) at broken ends. In the presence of the MRN complex, DNA-PK stimulated the cleavage of DNA ends by MRN and CtIP phosphorylation. These DNA-PK-mediated processes promote a sequential transition from NHEJ to HR.

In eukaryotic HR, the Rad51-ssDNA presynaptic complex plays a crucial role in homology search and strand exchange after DNA end resection, and Rad52 facilitates formation of the Rad51 presynaptic complex (Symington, 2016). ST-DNA curtain revealed that Rad52 reduces long-range DNA resection by inhibiting DNA end binding and translocation of Sgs1, which is a yeast helicase (Yan et al., 2019). The addition of Rad52 increased the dissociation of Sgs1 from the DNA end and prevented Sgs1 from binding to the DNA end. DNA-bound Sgs1 did not move in the presence of Rad52, indicating that Rad52 inhibits the motor activity of Sgs1.

### 5.2 Double-tethered DNA curtain

In the ST-DNA curtain, continuous hydrodynamic flow is necessary for stretching DNA. However, the flow not only exerts force to both DNA and proteins but also consumes a significant amount of sample. To circumvent these problems, two types of nano-patterns were fabricated, one for a diffusion barrier as in the ST-DNA curtain and the other for DNA attachment, which is pentagon-shaped and coated with antidigoxigenin (Gorman et al., 2010). The other end of DNA, at which digoxigenin is tagged, is tethered to the antidigoxigenin-coated pentagon pattern (Figure 5B), which is named as the double-tethered DNA curtain (DT-DNA curtain). In this geometry, DNA molecules remain stretched in the absence of flow, and protein-DNA interactions and protein motion on DNA can be observed without external force.

In eukaryotic mismatch repair, Msh2-Msh6 and Msh2-Msh3 complexes recognize single mismatches and multiple DNA mismatches, respectively (Kunkel and Erie, 2005). Using DT-DNA curtain, the molecular mechanism by which Msh2-Msh3 searches for DNA mismatches through genomic DNA was explored (Brown et al., 2016). DT-DNA curtain assay demonstrated that Msh2-Msh3 diffuses along DNA via 1D diffusion with both hopping and sliding to scan DNA. In the hopping mode, a protein transiently dissociates from DNA and reassociates into DNA, whereas a protein maintains its intimate contact with DNA in the sliding mode. Structurally, Msh2-Msh3 forms a ring structure and encompasses duplex DNA. Hence, it was expected that the protein slides along DNA. However, the diffusion coefficient of Msh2-Msh3 increased according to ionic strength, and competitor DNA rapidly dissociated Msh2-Msh3 from DNA. The transition of Msh2-Msh3 between neighboring DNA molecules was also observed. Taken together, these results demonstrate that Msh2-Msh3 hops on DNA and bypasses protein obstacles such as nucleosomes, presumably because of transient opening and closing of its ring structure.

R-loops are triple-stranded nucleic acids structures consisting of an RNA-DNA hybrid and a displaced ssDNA. R-loops play important roles in various biological reactions including immunoglobulin switching, chromosome segregation, and gene expression (Roy et al., 2008; Kabeche et al., 2018; Niehrs and Luke, 2020). However, abnormal accumulation of R-loops causes genomic instability (Hegazy et al., 2020). Recent studies report that m6A RNA methylation by METTL3-METTL14 plays a key role in R-loop resolution (Abakir et al., 2020). Interestingly, it was discovered that tonicity-responsive enhancer binding protein (TonEBP), a transcription factor that regulates cellular osmotic pressure, recognizes R-loops and contributes to R-loop resolution (Kang et al., 2021). ST-DNA curtain confirmed the colocalization of TonEBP and an R-loop that was inserted into lambda DNA at a specific location, showing the preferential binding of TonEBP to the R-loop. DT-DNA curtain demonstrated that TonEBP searches for R-loops using both 3D collision and 1D diffusion via sliding without helix rotation. This dual-mode search mechanism enhances R-loop finding through long genomic DNA. Biochemical assay clarified that TonEBP recognizes the displaced ssDNA of the R-loop.

DT-DNA curtain was also used to characterize the mechanistic properties of DNA translocases (Lee et al., 2012a; Lee et al., 2014). DNA translocases play important roles in DNA repair. The key reaction in HR is homology search and strand exchange of a presynaptic complex (Haber, 2018; Crickard et al., 2020). Rad54, which belongs to the Swi2/Snf2 family, is involved in the homology search process. DT-DNA curtain revealed the molecular function of Rad54 in homology search (Crickard et al., 2020). Rad54 bound to a Rad51-presynaptic complex translocated along duplex DNA, demonstrating that Rad54, as a molecular motor, guides the Rad51 presynaptic filament to donor DNA. Rad54 also unwound donor DNA and made bubble formation to facilitate the homology search.

### 5.3 Single-stranded DNA curtain

Most DNA repair processes require ssDNA as an intermediate, which interacts with repair enzymes such as recombinases. DNA curtain made of ssDNA, called ‘ssDNA curtain,’ was developed to probe the interactions between ssDNA and proteins. Long ssDNA is generated by rolling circle amplification from circular ssDNA annealed with a biotinylated primer. Because ssDNA forms secondary structures, eGFP-tagged RPA (RPA-eGFP) is coated on and stretches ssDNA to form an ssDNA curtain (Gibb et al., 2012) (Figure 5C).

ssDNA curtain has been applied to elucidate molecular mechanisms underlying HR. The homology search and strand exchange mechanism of the presynaptic complex was thoroughly scrutinized using ssDNA curtain. In the ssDNA curtain, the Rad51 presynaptic complex was formed by adding unlabeled Rad51 after free RPA-eGFP was washed out. While unlabeled Rad51 replaced RPA-eGFP, eGFP fluorescence disappeared and ssDNA became more flexible because Rad51 binding elongated ssDNA (Figure 5C). Qi *et al.* incubated fluorescently labeled duplex DNA containing microhomology (<15 bp) in the middle. The authors found that 8-bp or longer microhomology stably binds to the Rad51 filament, implying that at least an 8-nt match is required for strand exchange (Qi et al., 2015). The dissociation rate of microhomology decreased in a stepwise manner for every 3 nts increase in length, indicating that each 3-nt step has the same energy and that strand exchange occurs every 3 nts because the Rad51 monomer takes three nucleotides structurally. Furthermore, this 3-nt stepping behavior is evolutionarily conserved from *E. coli* RecA to human Rad51 and Dmc1 (Lee et al., 2015). In particular, Dmc1, a meiotic-specific recombinase, is more tolerant to mismatches than Rad51, suggesting that genetic diversity occurs during meiosis. Zhao *et al.* pried into the effect of the tumor suppressor complex BRCA1-BARD1 on the formation of presynaptic filaments during HR using an ssDNA curtain (Liang et al., 2016). BRCA1-BARD1 enhanced the binding of microhomology-containing duplex DNA to Rad51 presynaptic filaments. However, BRCA1-BARD1 did not influence the binding sites and lifetime of DNA. These results suggest that BRCA1-BARD1 increases the association rate and promotes presynaptic complex formation. ssDNA curtain was also used for studying RecQ helicase, which removes untimely formed Rad51 filaments and promotes proper strand invasion (Branzei and Foiani, 2007). RecQ translocation was visualized on both RPA-coated ssDNA and Rad51 filaments. RecQ displaced both RPA and Rad51 from ssDNA. Nevertheless, RecQ did not actively unwind duplex DNA, and hence RecQ was mostly stalled at a heteroduplex structure in the ssDNA curtain. The molecular function of Rad51 paralogs was also determined. Taylor *et al.* discovered that RFS-1/RIP-1, which is a Rad51 paralog in *C. elegans*, promotes HR by remodeling the Rad51 presynaptic filament (Taylor et al., 2016). When the unlabeled Rad51 filament was completely formed, re-injection of RPA-eGFP replaced Rad51 again in the absence of ATP and then eGFP fluorescence was recovered. However, RFS-1/RIP-1 suppressed RPA-eGFP replacement, meaning that the paralogs stabilize the Rad51 filament regardless of nucleotide cofactors. The authors showed that RFS-1/RIP-1 caps the 5′ end of the Rad51 filament and suppresses disassembly of Rad51 from the 5’ end.

Unregulated HR induces unwanted recombination, threatening genome stability. Anti-recombinases down-regulate HR (Karpenshif and Bernstein, 2012). Srs2, as an anti-recombinase, detaches Rad51 from ssDNA and suppresses HR (Antony et al., 2009). In the ssDNA curtain, the addition of *S. cerevisiae* Srs2 to Rad51 presynaptic filaments induced the association of RPA-eGFP from the 3′ end of ssDNA because the translocation of Srs2 along ssDNA from the 3′ to 5′ end dislodges Rad51 (Kaniecki et al., 2017) (Figure 5C). Fluorescently labeled Srs2 displayed multiple photobleaching steps. The increase in Srs2 concentration elevated the processivity and speed of Srs2 translocation. These results imply that Srs2 acts as a multimeric complex. Interestingly, the RPA cluster and Srs2 colocalized, showing that Srs2 is recruited to the RPA cluster in the Rad51 presynaptic complex. Srs2 also removes mispaired intermediates during strand exchange in HR (Niu and Klein, 2017). In the ssDNA curtain, Srs2 translocated from the 3′ to 5’ end along RPA-coated ssDNA and displaced RPAs (De Tullio et al., 2017). Srs2 also removed Rad52 bound to RPA-coated ssDNA (Nimonkar et al., 2009). These data demonstrate that Srs2 dismantles Rad52-induced mispairing intermediates and suppresses improper HR.

## 6 DNA tightrope and skybridge

**FIGURE 6:**
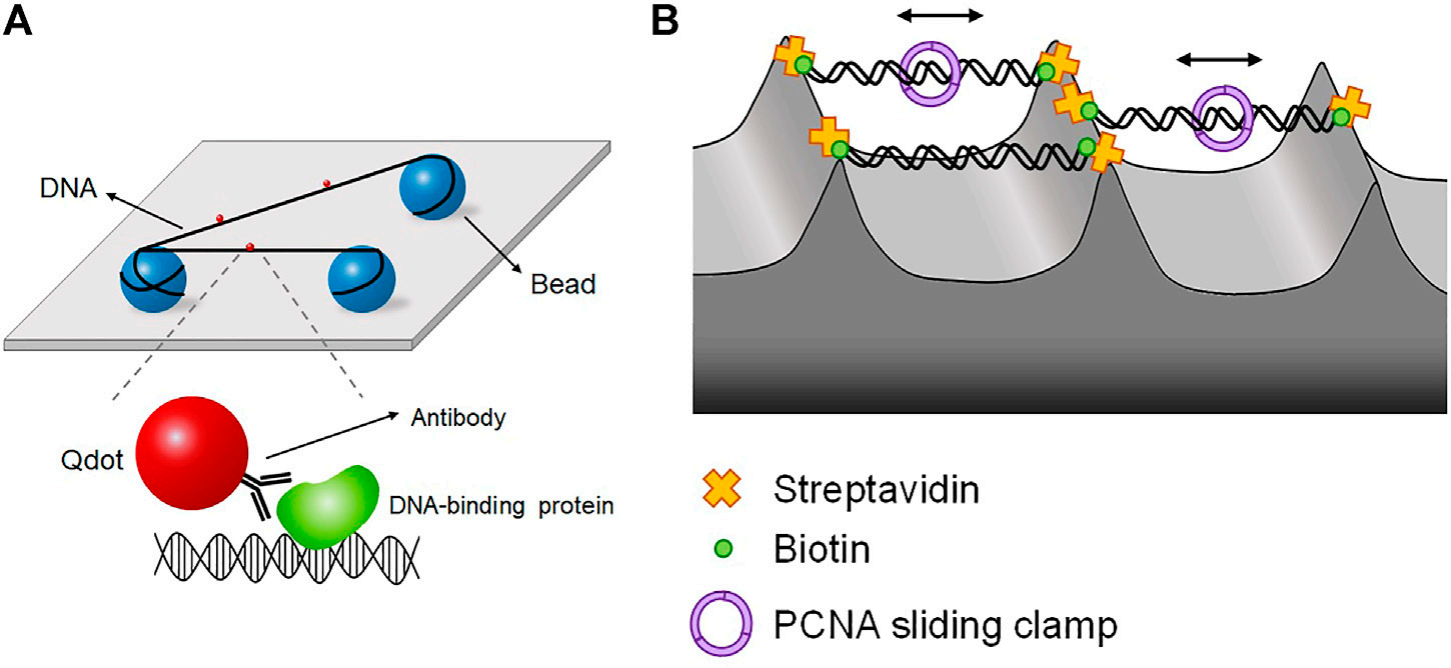
DNA tightrope and DNA skybridge. **(A)** DNA tightrope assay, in which DNA molecules are suspended between micrometer-sized beads and placed far above the surface, preventing the surface effect. Proteins are conjugated with Qdot, which is imaged by oblique angle excitation by laser instead of TIRFM. **(B)** DNA skybridge technique, in which biotinylated DNA molecules hang between streptavidin-coated apices of 3D microstructures, which are fabricated by photolithography and chemical etching. PCNA diffusion is observed in real time using DNA skybridge.

When DNA is placed close to a surface, the surface may interfere with protein-DNA interactions. This surface effect is a big challenge for single-molecule imaging techniques based on surface-tethering. This problem can be circumvented by floating biomolecules far above the surface. In DNA tightrope assay, DNA molecules are suspended between micrometer-sized beads like tightropes (Kad et al., 2010; Hughes et al., 2013; Kong et al., 2016; Springall et al., 2018; Lee et al., 2020; Jang et al., 2021) (Figure 6A). Beads are coated with positively charged chemicals such as poly-l-lysine and then spread on the surface. Negatively charged DNA molecules are randomly attached to beads. In the presence of flow, DNA molecules are stretched, and the other end of DNA is tethered to another bead to form DNA tightropes. Once DNA tightropes are formed, flow is no longer necessary. In this geometry, DNA sequence and orientation are not well defined. Target proteins are labeled with Qdot. As DNA is far above the surface, TIRFM is not applicable. Instead, the excitation laser is obliquely incident and propagates in the opposite direction to the CCD camera. This oblique angle excitation can reduce the background from the laser.

Using the DNA tightrope assay, Bennett Van Houten and his colleagues studied the NER pathway in *E. coli* (Kad et al., 2010; Hughes et al., 2013; Springall et al., 2018). In *E. coli* NER, the UvrAB complex finds DNA lesions, and UvrC makes incisions around a lesion (Sancar and Rupp, 1983; Verhoeven et al., 2000; Goosen and Moolenaar, 2008; Kad et al., 2010). The authors’ research interest was the damage search mechanism of *E. coli* NER proteins through DNA. UvrA and UvrB were labeled with differently colored Qdots and fluorescently imaged in DNA tightropes (Kad et al., 2010). UvrA and UvrB were colocalized at DNA lesions, ensuring UvrAB complex formation. In addition, the authors checked the dimerization of UvrB with differently labeled UvrB monomers in the presence of unlabeled UvrA. The UvrAB complex exhibited three distinct types of motion along DNA—one-dimensional diffusion (61%), unidirectional movement (19%), and pause (19%)—showing that the UvrAB complex has several distinct conformational states, called ‘conformational flexibility’, during lesion search. The authors next looked into the interaction between UvrB and UvrC (Hughes et al., 2013). They demonstrated that UvrC forms a complex with UvrB in solution and facilitates the binding of UvrB to DNA, although UvrC alone can bind DNA. UvrBC diffused along DNA, and the increase in ionic strength raised the diffusion constant of UvrBC, indicative of diffusion via hopping. They proposed that diffusion of the UvrBC complex brings UvrC to a pre-incision complex at a lesion, after which UvrC makes incisions around the lesion. The authors then asked how Uvr proteins form multimeric complexes and which multimers identify DNA lesions with lesion-containing DNA (Springall et al., 2018). In the tightrope assay, each Uvr protein was labeled with differently colored Qdot and visualized with multi-color imaging. The authors showed that UvrBC can identify DNA lesions, although UvrAB serves as the primary lesion-sensing complex. In addition, it was revealed that UvrA, UvrB, and UvrC could assemble into a trimeric complex. DNA tightrope assay was extended to the eukaryotic NER pathway including UV-DDB, a complex of DDB1 and DDB2, which preferentially detects CPD that is not sensed by XPC-RAD23B due to small DNA distortion (Sugasawa, 2009). UV-DDB along with the E3 ubiquitin ligase Cullin4 ubiquitinates UV-DDB itself and XPC-RAD23B to transfer CPD from UV-DDB to XPC-RAD23B. The tightrope assay revealed that UV-DDB performs a 3D search mechanism rather than 1D diffusion along DNA (Ghodke et al., 2014). Furthermore, UV-DDB displayed three distinct dissociation rates at DNA lesions. Such heterogeneity in damage sensing kinetics implied that UV-DDB undergoes discrete conformational changes to be stabilized at lesions.

Another approach for excluding the surface effect is ‘DNA skybridge,’ in which DNA molecules hang between three-dimensional structures on a quartz slide that are fabricated by photolithography and chemical etching (Kim et al., 2019) (Figure 6B). On apices, streptavidin is deposited by PDMS stamping, and DNA molecules are anchored on the apices via biotin-streptavidin linkage. Fluorescence excitation is conducted by a laser light sheet with slant incidence, which makes an interference pattern from the periodically formed skybridge structures. In this configuration, DNA is randomly oriented because both ends of DNA are labeled with biotin. Using DNA skybridge, the motion of PCNA, which is a sliding clamp, was monitored in real-time (Kim et al., 2019) (Figure 6B). DNA skybridge was also used for studying human Mlh1-Pms2, which plays a role in excision during MMR. Mlh1-Pms2 alone did not show any diffusive motion but stably diffused along DNA when Msh2-Msh6, a mismatch sensor, was present. These results indicate that Msh2-Msh6 provides a platform for the diffusion of Mlh1-Pms2 for downstream excision (London et al., 2021).

## 7 Hybrid single-molecule force-fluorescence techniques

Force spectroscopy is a unique, powerful single-molecule arsenal that can mechanically manipulate single biomolecules, allowing the study of their mechanical properties, conformational transitions, and possible mechanisms underlying biomolecular interactions (Moffitt et al., 2008; Neuman and Nagy, 2008; De Vlaminck and Dekker, 2012; Heller et al., 2014; Sarkar and Rybenkov, 2016; Carlos et al., 2021). In optical/magnetic tweezers, a target biomolecule is trapped between the flow cell bottom and a polystyrene/magnetic bead. Tension and/or torsion is then applied to the bead by (electro)magnetic fields, and the resultant positional change is tracked in real-time to determine the structural/conformational change of the target molecule. Single-molecule tweezers can explore the stretching and supercoiling properties of nucleic acids, (un)folding transitions and energy landscapes of some special nucleic acid structures like G4 or RNA pseudoknot, and interactions between DNA and proteins with high accuracy (Chen et al., 2007; Moffitt et al., 2008; De Vlaminck and Dekker, 2012; Heller et al., 2014; Carlos et al., 2021). Such optical and magnetic tweezers techniques have also provided valuable information and insights into DNA damage repair, such as RecA-driven DNA HR in real time, stretching/torsional properties of RecA-DNA filaments and their formation kinetics, and Rad51-driven twisting events of DNA (Hegner et al., 1999; van der Heijden et al., 2008; Arata et al., 2009; van Loenhout et al., 2009; Lipfert et al., 2010; Andrea Candelli et al., 2013). A unique hybrid method combining optical and magnetic tweezers could bring together a RecA-DNA filament and normal naked DNA and investigate their homology sampling and recognition (De Vlaminck et al., 2012).

**FIGURE 7:**
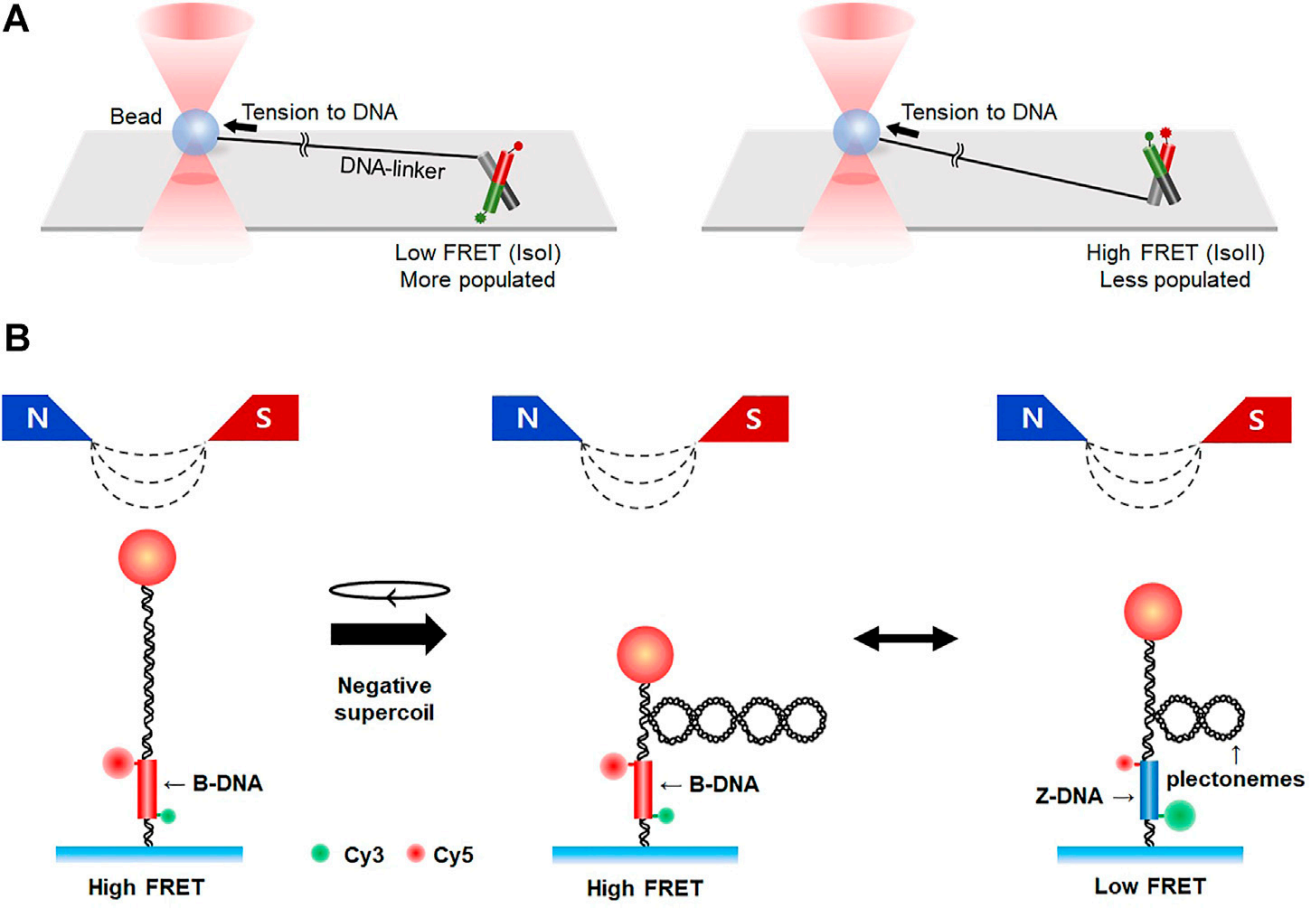
Hybrid methods combining single-molecule fluorescence imaging with force spectroscopy. **(A)** Integration of smFRET with optical tweezers. Conformational dynamics of HJ are measured by smFRET in the presence of tension, which is exerted to HJ by optical tweezers. Tension to HJ induces a larger population of IsoI conformers (left) than IsoII (right). **(B)** Hybrid system combining smFRET and magnetic tweezers. Negative supercoiling is applied to DNA by magnetic tweezers, resulting in the formation of plectonemes. When B-to Z-transition occurs, the number of plectonemes is reduced and FRET efficiency changes from high to low.

Combining fluorescence imaging methods with single-molecule tweezers provides an additional degree of freedom in the observation of unrevealed biological phenomena in bulk. Arai *et al.* were able to control the buckling curvature of a single actin filament and measure its breakage force using fluorescence imaging combined with dual-trap optical tweezers (Arai et al., 1999). smFRET merged with optical tweezers was able to estimate subtle changes in the structural transition kinetics of unusual DNA structures like HJ, showing that the IsoI conformer was more populated than the IsoII conformer under tension (Hohng et al., 2007) (Figure 7A). smFRET was also combined with magnetic tweezers (Figure 7B). This hybrid method enabled real-time observation of the B- to Z-DNA transition under tension/torsion, which is difficult to measure with individual methods because the transition involves subtle changes in optical chirality and thus has a very small distance change along the DNA (Lee M. et al., 2010; Kim et al., 2021). Z-DNA can be formed in alternating pyrimidine-purine repeats such as TG-repeats, in which DNA double-strand breaks frequently occur. The fragile sites are repaired by microhomology-mediated end-joining (Xie et al., 2019). Magnetic tweezers combined with smFRET are unique in that it measures small rotational motions while applying torsion and tension simultaneously, which should be a good addition to the toolset for studying DNA damage repair. DNA curtain assay integrated with optical tweezers directly showed how the binding of *E. coli* RNA polymerase to DNA is influenced by applied tension (Lee et al., 2012b). More sophisticated single-molecule systems have also been established such as a combination of single/dual-trap optical tweezers, fluorescence imaging, and microfluidics (Galletto et al., 2006; Hilario et al., 2009; Candelli et al., 2014; Brouwer et al., 2018). Using these combined techniques, RecA assembly on dsDNA and Rad51 (dis)assembly on dsDNA/ssDNA were directly visualized. Furthermore, Rad51 filaments on ssDNA were shown to have two distinct conformational states in the ATP-bound state and, upon hydrolysis, convert into a disassembly-competent ADP-bound state, demonstrating that the Rad51-ssDNA interaction dynamically changes according to ATP/ADP cycles (Brouwer et al., 2018).

## 8 Live-cell imaging

Most single-molecule fluorescence imaging is conducted *in vitro* with purified proteins under well-defined biochemical environments. Although *in vitro* single-molecule imaging reveals a plethora of molecular details, it still has innate limitations of *in vitro* systems, which do not completely resemble intracellular situations. Many attempts have been made to perform fluorescence imaging inside a cell at the single-molecule level. We briefly introduce several *in vivo* imaging techniques for DNA repair.

DSB induced by ionizing radiation was visualized using GFP-tagged proteins related to DNA damage repair in a living cell (U2OS) (Jakob et al., 2009). 53BP1, as a damage response factor, facilitates cluster formation for DNA damage repair. Tracking GFP-53BP1 demonstrates that damaged chromatin slowly diffused because chromosomal regions containing DSB were clustered with other repair proteins (Aten et al., 2004; Jakob et al., 2009). Live-cell imaging also determined which repair pathway is selected according to the cell cycle (Karanam et al., 2012). Rad52 forms foci at DSB sites to commit homology-dependent repair, and 53BP1 protein is a canonical marker for DSBs. Rad52 and 53BP1 were tagged with different fluorescent proteins. In each phase of the cell cycle, the proportion of Rad52 and 53BP1 foci was measured in a living cell, verifying that NHEJ is the major repair pathway for DSBs in G1 and G2, whereas HR is dominant in the middle of S-phase. On the other hand, fluorescence imaging of histone proteins in a damaged cell revealed that DNA damage can change the structure or composition of chromatin (Hauer et al., 2017). Damaged chromatins lost histones and became mobile. This high mobility of damaged chromatins increased HR efficiency.

Kensuke Otsuka and Masanori Tomita developed a new live-cell imaging tool using custom-designed plasmids called ‘Focicle’ (‘foci’ + ‘cell cycle’), which is a tri-cistronic cassette encoding the fluorescent 53BP1 foci-forming region and two cell-cycle indicators (hCdt1 and hGmnn) (Otsuka and Tomita, 2018). To build Focicle knock-in cell lines, they utilized CRISPR/Cas9-mediated targeting. Focicle facilitated measurement of the formation of 53BP1 foci and kinetics of DSB repair in a living cell after radiation exposure. Moreover, the authors demonstrated that Focicle probes can be cell-cycle indicators that identify the cell-cycle state (e.g., arrest/progression).

Finally, Mine-Hattab et al. showed that single-molecule microscopy facilitates visualization of the dynamic behavior of Rad52 in living cells containing DSBs (Mine-Hattab et al., 2021). Particle-tracking showed that Rad52 molecules accumulate to form foci at broken DNA, and the diffusion of Rad51 in the foci is slower than that of free Rad52. Interestingly, the diffusion coefficient of Rad52 increased while Rad52 entered and escaped from the foci, suggesting that its diffusion behavior is determined by the environment. When multiple DSBs occurred, diffusion of individual Rad52 molecules accelerated, leading to an increase in Rad52 foci. Such physical behavior of Rad52 shows that Rad52 molecules explore their foci like liquid droplets around damaged DNA during DSB repair.

## 9 Perspectives

Single-molecule fluorescence imaging techniques are powerful tools for probing biological phenomena in minute detail, both *in vivo* and *in vitro*. Direct and intuitive evidence from single-molecule imaging provides a better understanding of biomolecular interactions that would be veiled by the ensemble effect of bulk assays. In this paper, we surveyed broad applications of single-molecule imaging for DNA damage repair. DNA damage repair is essential for maintaining genomic integrity and is closely associated with numerous human diseases. Repair processes involve complicated signaling pathways and the cooperative activity of many repair proteins. Single-molecule fluorescence techniques reveal the molecular mechanisms and bases underlying repair pathways, leading to a better understanding of human diseases and progress in developing therapeutics. In addition to single-molecule techniques, other types of bio-physico-chemical methods with high spatiotemporal resolution have been developed, such as cryo-electron microscopy and high-speed atomic force microscopy. With the use of these techniques, single-molecule fluorescence imaging methods will more comprehensively expand our understanding of complex DNA repair signaling.
